# Coronal Pulpotomy Technique Analysis as an Alternative to Pulpectomy for Preserving the Tooth Vitality, in the Context of Tissue Regeneration: A Correlated Clinical Study across 4 Adult Permanent Molars

**DOI:** 10.1155/2015/916060

**Published:** 2015-05-17

**Authors:** Raji Viola Solomon, Umrana Faizuddin, Parupalli Karunakar, Grandhala Deepthi Sarvani, Sevvana Sree Soumya

**Affiliations:** Department of Conservative Dentistry and Endodontics, Panineeya Institute of Dental Sciences and Research Center, Kamala Nagar, Dilsukhnagar, Telangana 5000060, India

## Abstract

*Aim*. (1) The aim of the clinical study revolves around the accurate diagnosis, proper case selection, and the management of acute irreversible pulpitis in permanent molars with closed apices using conservative and economical treatment modalities like vital pulpotomies with regenerative approaches over conventional root canal procedures. (2) To evaluate the use of autologous substances such as platelet concentrates and calcium silicate based materials in promoting the healing and regeneration of the inflamed pulp.* Summary*. Vital pulpotomy was performed on 5 carious involved, permanent molars diagnosed with acute irreversible pulpitis in 17- to 22-year-old patients. Taking into consideration the patient's age and the condition of the underlying pulp tissue, PRF pulpotomy was planned in view of preserving the vitality of the intact radicular pulps. Regenerative procedures with second generation blood matrices were chosen to encourage the recovery of the inflamed pulps. The systematic follow-up examinations performed at 3, 6, 9, 12, 18, 22, and 24 months revealed a successful clinical and radiological outcome. Within the limits of the present clinical study and correlating the success across the treated clinical cases, we safely conclude the potential scope of regenerative pulpotomy approaches in acute irreversible pulpitis in adult permanent teeth.

## 1. Introduction

The dental pulp is a soft connective tissue confined within the hard walls of the dentin which plays an important role in the prognosis of the tooth, which is often ignored. Exposure of the pulp due to various reasons like caries, fractures, cracks, or an open restoration margin often results in inflammation of the pulp which can subsequently lead to pulpal death if not treated at the earliest [1]. Therapeutics of dental pulp diseases include vital pulp therapies like direct and indirect pulp capping, pulpotomy in the initial stages, or pulpectomy if the lesion presents in its later stages. Preservation of pulpal vitality is of paramount importance as the vital functioning pulp is capable of initiating several important functions like formation of dentin, providing nutritive support to the tooth, enabling a defensive function, and possessing a unique reparative capacity [2]. Hence, it is beneficial to preserve the vitality of the pulp rather than to replace it with an inert root filling material.

Pulpotomy is a vital pulp therapy in which the coronal portion of the pulp is removed surgically and the remaining radicular pulp is preserved intact. Over the remaining radicular pulp tissue, a suitable material is placed which has the potential to protect the pulp from further insult and initiate healing and repair [3].

The rationale behind pulpotomy procedures is based on the ability of the remaining radicular pulp to Recover following the removal of the infected coronal pulp tissue and placement of a suitable medicament [4]. Various materials have been advocated for use in pulpotomy procedures based on their important properties such as biocompatibility, sealing ability, and antimicrobial efficacy when placed in contact with the inflamed pulp. MTA is one of the most commonly used and researched material for such purposes with successful clinical outcomes. However, due to certain inherent drawbacks of MTA, there is a need for the development of newer materials that addresses the requirements of pulpotomy therapies and that can overcome the challenges associated with MTA [5].

Biodentine is a calcium silicate based material that has fetched attention in recent years and became commercially available in 2009 (Septodont, http://www.septodontusa.com/). It was initially and specifically designed as a “dentin replacement” material. Biodentine is primarily formulated using the MTA-based cement technology with improvement in some properties of these types of cements, such as physical qualities and handling [6].

Biocompatibility of a dental material is another major factor that should be emphasized upon, specifically when it is used in vital pulp therapy. In a performed animal study, the authors [7] assessed the pulpal response of primary pig teeth against Biodentine when used as a pulp capping as well as a pulpotomy material after 7, 28, and 90 days. Their results revealed that Biodentine has bioactive properties, encourages hard tissue regeneration, and provokes no signs of moderate or severe pulp inflammation responses. They further noted through their investigations that the material had the ability to maintain a successful marginal integrity due to the formation of hydroxyapatite crystals at the surface which improved its sealing ability.

Inspite of the growing improvements in material sciences, research still documents the mild to moderate cytotoxic effects of various biomaterials used for pulpotomies, when they are placed in direct contact with the pulp tissue. Hence, there is a constant need for biologically based autologous materials to neutralize the side effects, if any, due to synthetic based biomaterials, to reduce the pulpal inflammation and to promote faster healing [8]. Platelet rich fibrin is a second generation platelet concentrate introduced by Choukroun et al. It is strictly autologous and helps to release the growth factors necessary for the regeneration of dentin pulp complex thereby accelerating the healing process [8, 9].

The case series presented describes the management of 5 pulpally involved carious human adult permanent molars with established acute irreversible pulpitis. The clinical and the radiological outcomes of the treated cases are cross correlated over systematic follow-up evaluations of all the 5 cases to gauge the prognosis of the treatment performed using second generation platelet concentrates (PRF) and a new calcium silicate based material (Biodentine) for coronal pulpotomy techniques.

## 2. Case Report

A 22-year-old female patient reported to the Department of Conservative Dentistry and Endodontics with a chief complaint of acute pain and with a request for dental care in the lower right back tooth region. The patient's symptom was confirmed with spontaneous and deep intense pain lingering even after the removal of the thermal stimulus. The response was reproduced on EPT test. The complete dental history was recorded with emphasis on the history of present illness. On extra oral examination, there was no swelling or tenderness with respect to that region.

**Figure 1:**
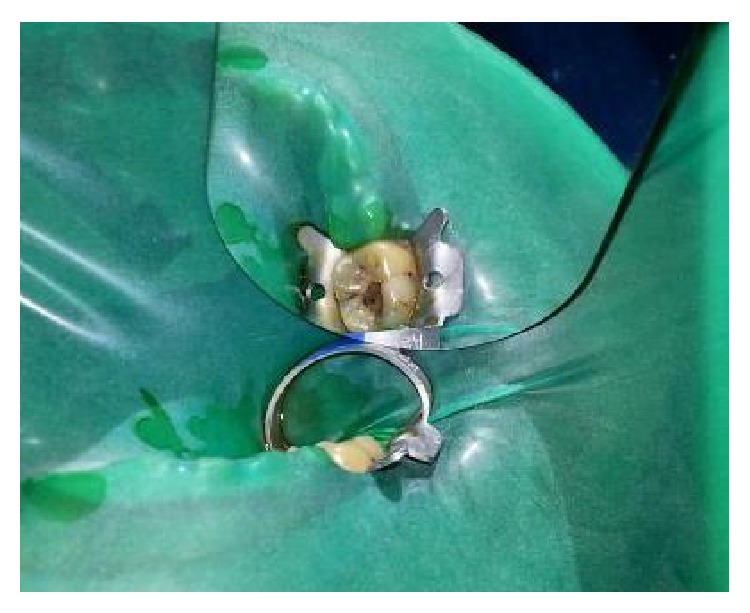
Preoperative photograph.

On clinical examination, there was a deep carious lesion involving the occlusal surface (Figure 1). There was no tenderness on percussion and no associated sinus opening adjacent to the tooth. On radiographic examination, the RVG image revealed a deep carious lesion involving the enamel, dentin, and pulp in the mandible right permanent first molar (Figure 7). Based on the clinical, radiographic, and pulp sensibility examinations, the diagnosis was established as symptomatic irreversible pulpitis.

The treatment modality of coronal pulpotomy using PRF and a calcium silicate based cement, namely, Biodentine, was explained to the patient as an alternative to the conventional root canal treatment. The written consent was obtained from the patient. The medical examination and tests for the bleeding time, clotting time, and platelet count were performed and were found to be in normal range.

PRF was prepared by drawing blood into a 10 mL test tube without the addition of an anticoagulant. Hence, to prevent the blood from coagulating after coming in contact with the glass tube, it was centrifuged immediately using a table top centrifuge at 3000 rpm for 15 mins.

The product thus obtained consisted of the three layers: the top most layer of acellular platelet poor plasma, the middle layer of platelet rich fibrin, and the bottom most layer of red blood corpuscles. The PRF was segregated and was squeezed to form a membrane (Figures 3 and 4).

**Figure 2:**
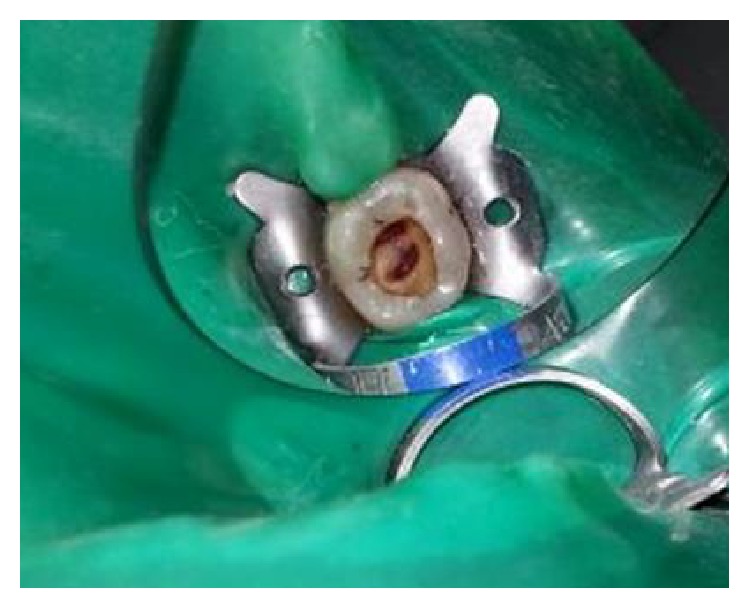
Access opening for pulpotomy procedure.

**Figure 3:**
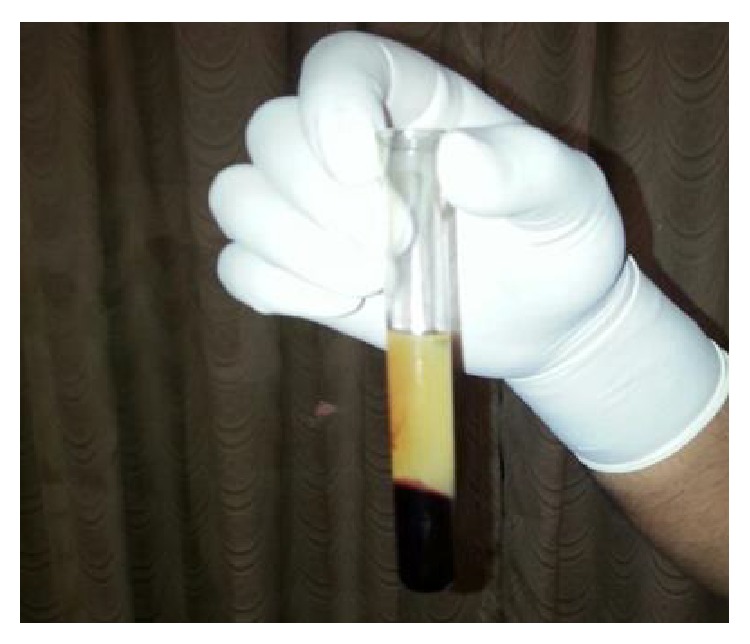
Preparation of platelet rich fibrin.

**Figure 4:**
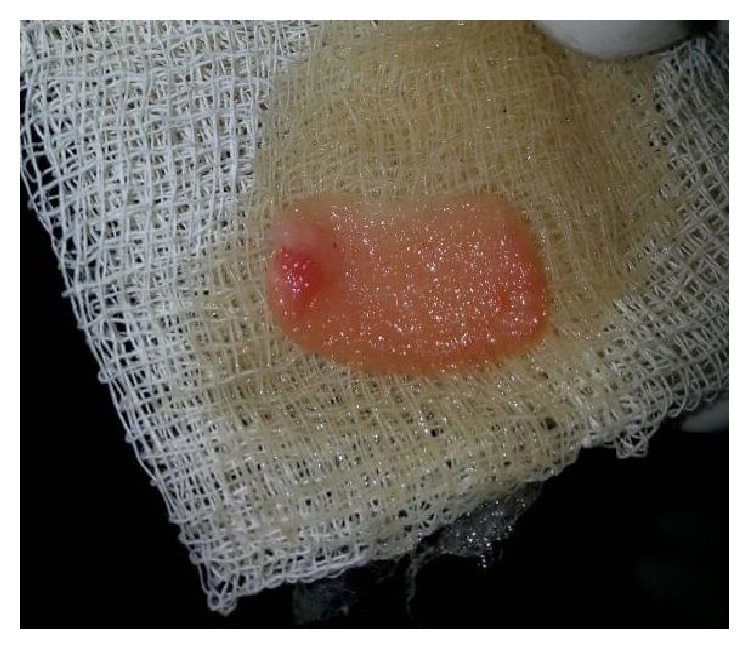
PRF membrane.

**Figure 5:**
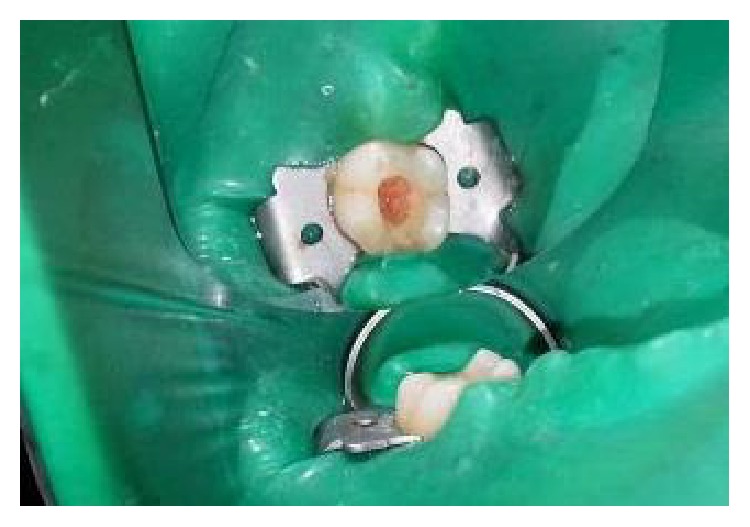
Placement of platelet rich fibrin over the radicular pulp.

**Figure 6:**
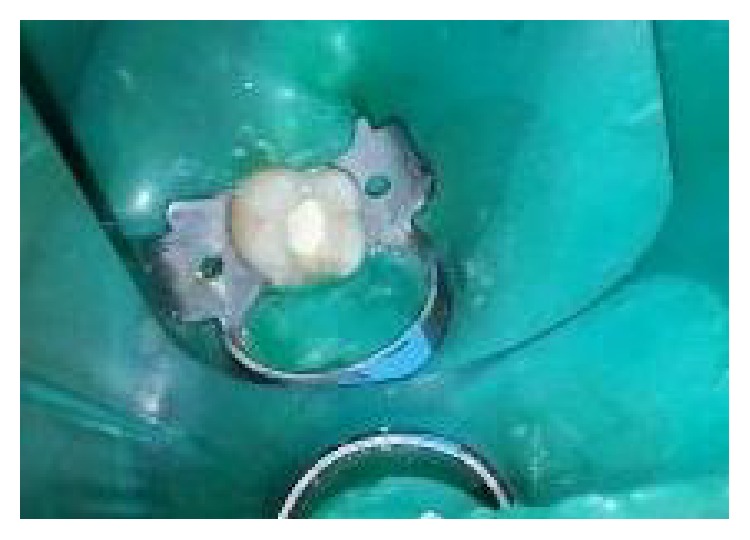
Placement of Biodentine over PRF membrane.

**Figure 7:**
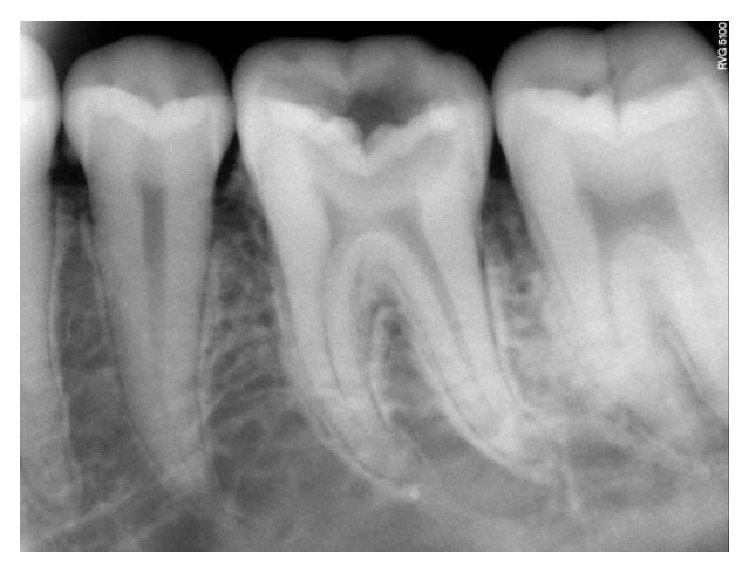
Preoperative RVG.

The tooth was anesthetized with an inferior alveolar nerve block using Lignocaine 2% with adrenaline and rubber dam isolation was achieved. Access to the carious lesion was gained and pulpotomy was performed using a high speed airotor hand piece and the coronal pulp tissue was removed till the pulpal floor (Figure 2). Hemostasis was attained using cotton pellets moistened with saline. The PRF membrane obtained after centrifugation of the patient's own blood withdrawn from the median cubital vein was placed over the exposed pulp stumps (Figure 5). Biodentine was placed over PRF to an approximate thickness of 2 mm and the final restoration was placed using direct composite restorative resin (Figure 6). Digital radiographs were taken and the patient was recalled after one day and evaluated for the presence of pain.

The identical procedure was performed in the remainder three patients diagnosed with similar pulpal pathology (Figures 14 [Fig fig15] [Fig fig16] [Fig fig17]–18, 25 [Fig fig26] [Fig fig27]–28, and 32 [Fig fig33] [Fig fig34] [Fig fig35]–36). In one of the clinical cases treated, in a male patient, of age 22 years, two permanent molars (first and second lower left molars) were subjected to the designated treatment plan (Figures 14–17).

## 3. Followup

**Figure 8:**
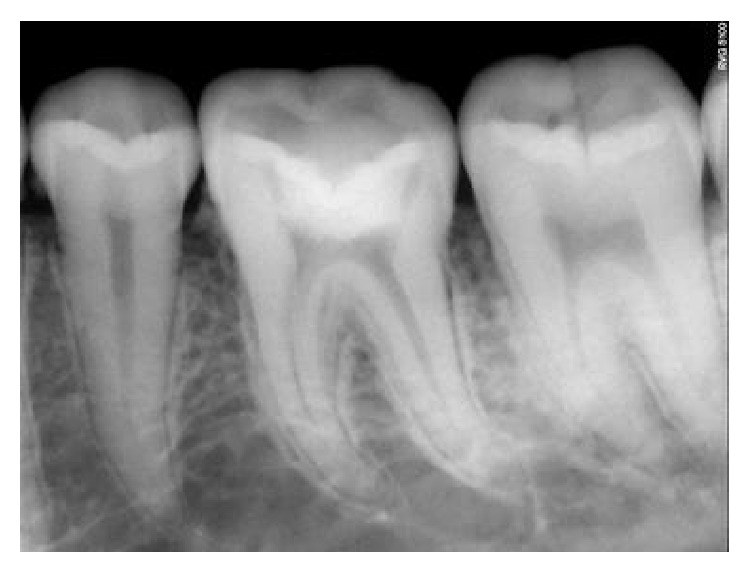
Immediate postoperative RVG.

**Figure 9 fig9:**
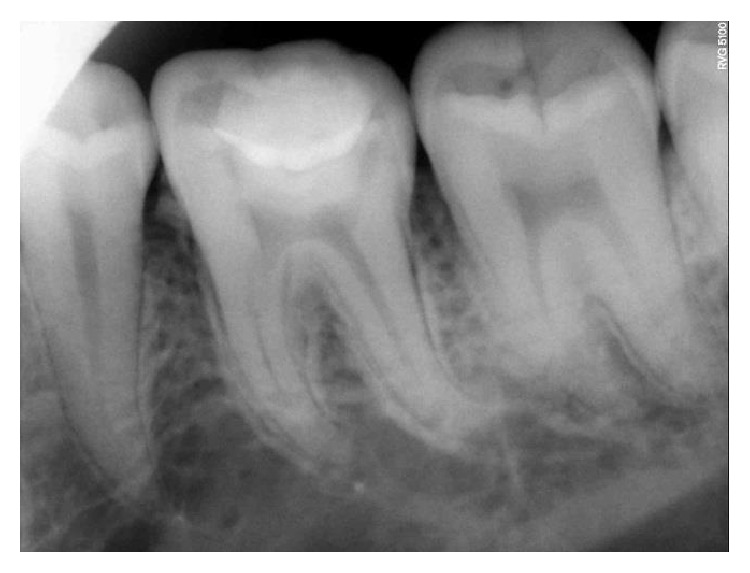
6-month followup RVG 46.

**Figure 10 fig10:**
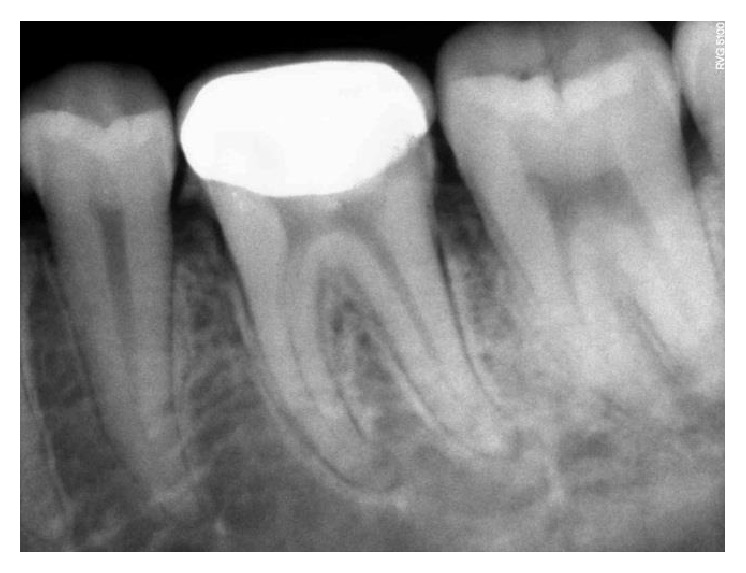
12-month followup RVG 46.

**Figure 11 fig11:**
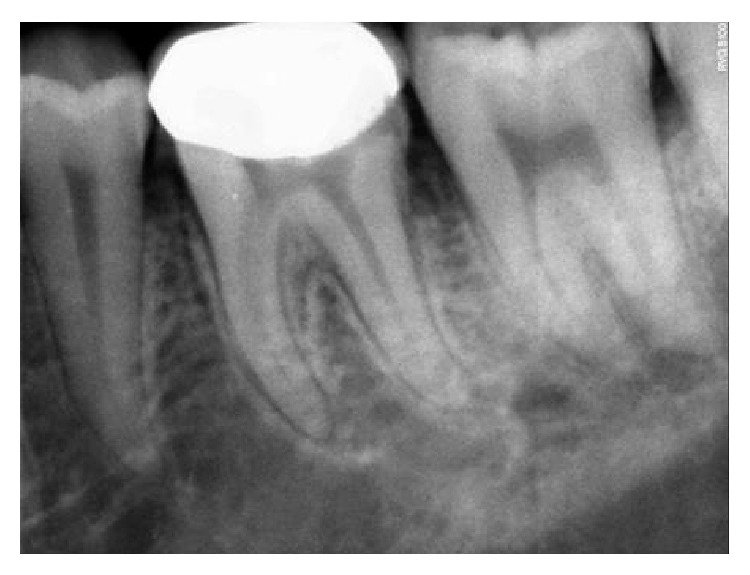
18-month followup RVG 46.

**Figure 12 fig12:**
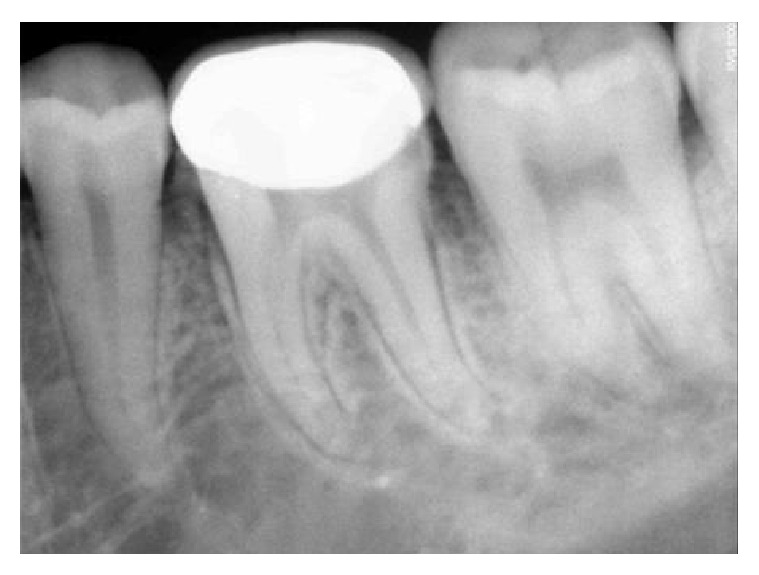
22-month followup RVG 46.

**Figure 13:**
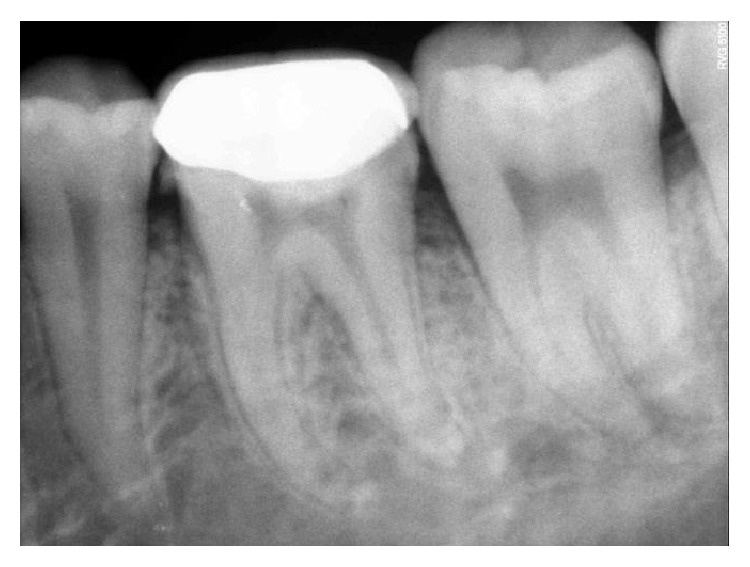
24-month followup RVG 46.

**Figure 19:**
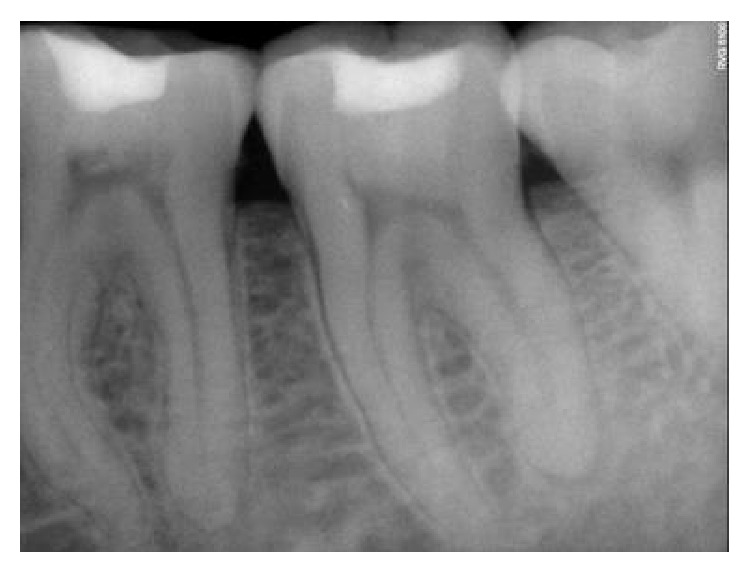
3-month followup RVG: 36, 37.

**Figure 20 fig20:**
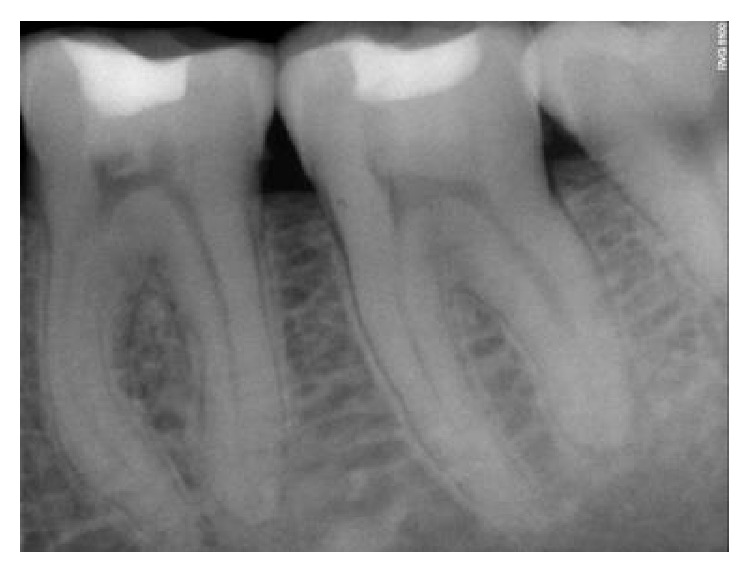
6-month followup RVG: 36, 37.

**Figure 21 fig21:**
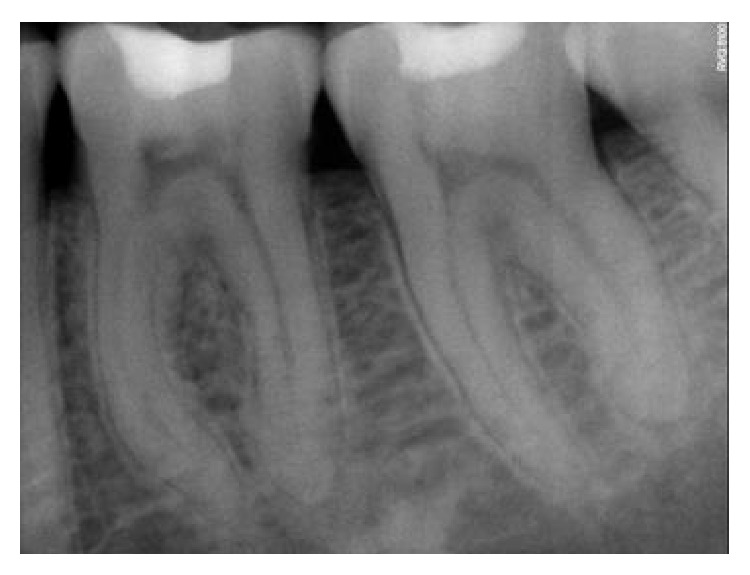
9-month followup RVG: 36, 37.

**Figure 22 fig22:**
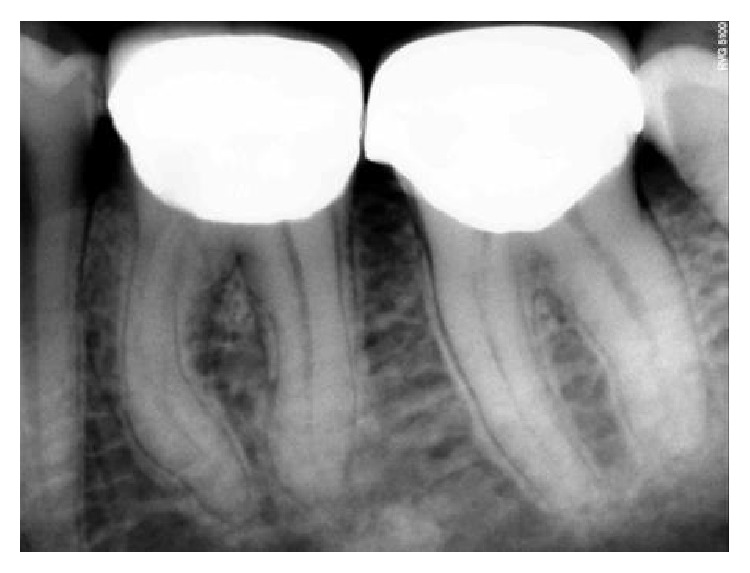
12-month followup RVG: 36, 37.

**Figure 23:**
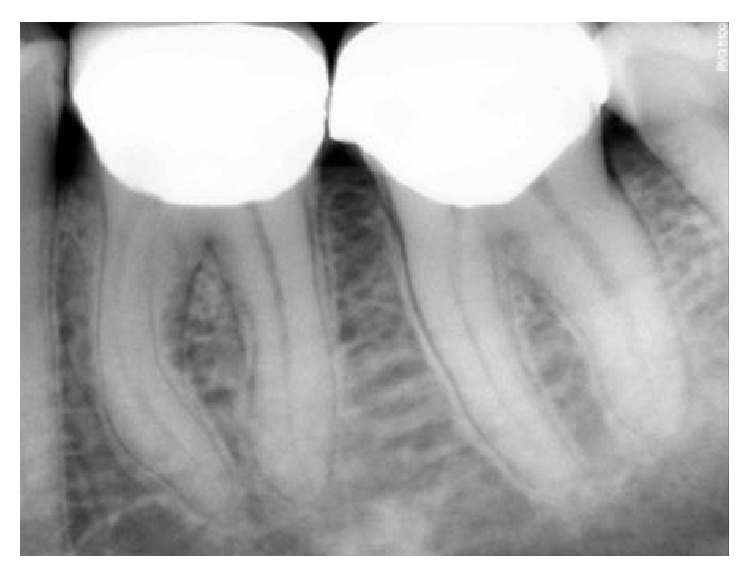
18-month followup RVG: 36, 37.

**Figure 29:**
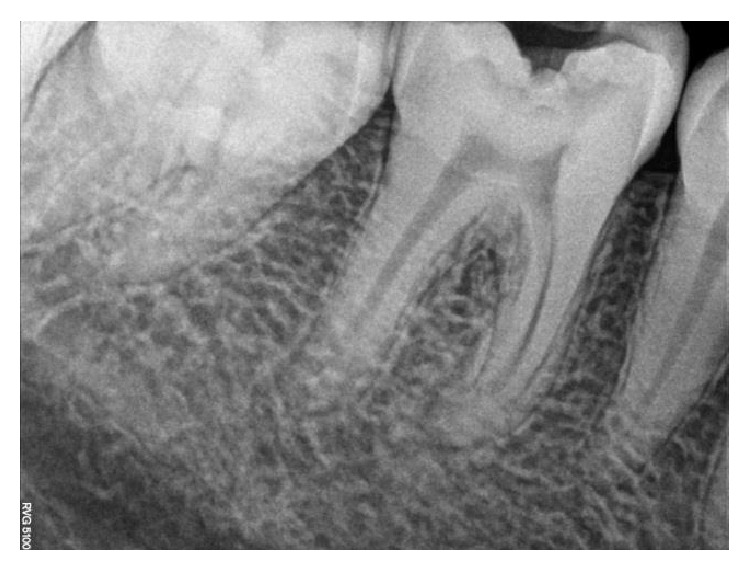
Preoperative RVG 36.

**Figure 30 fig30:**
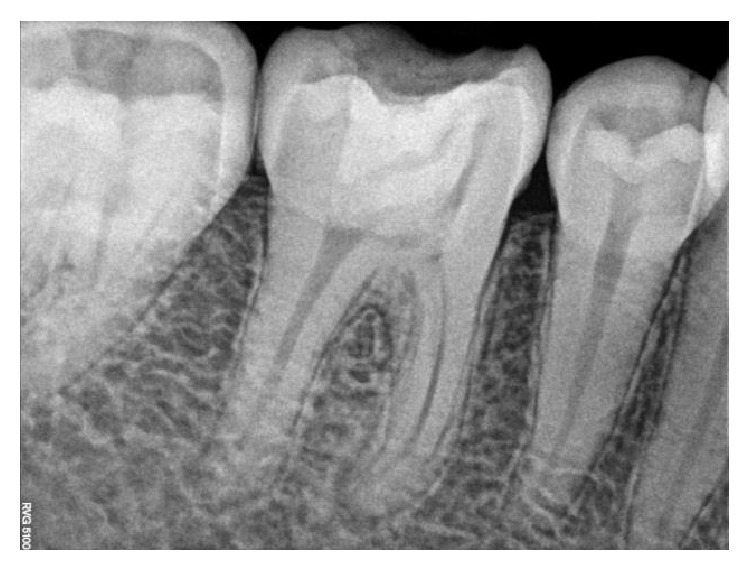
6-month followup RVG 36.

**Figure 31:**
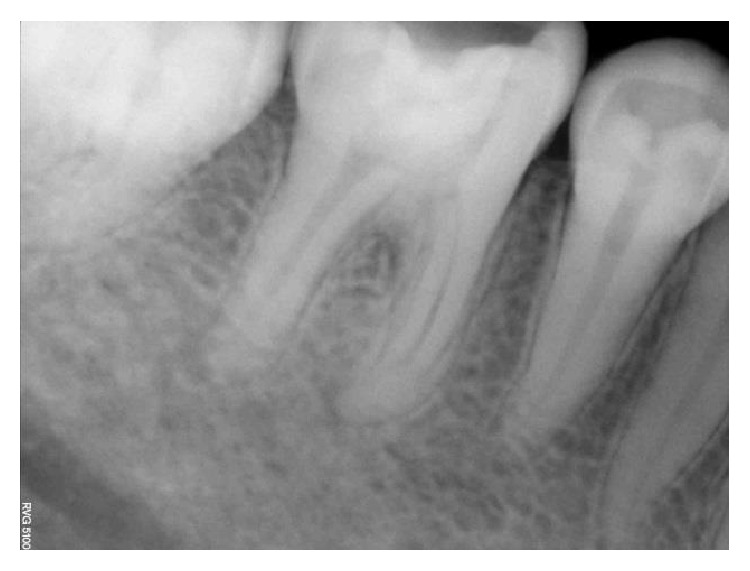
9-month followup RVG 36.

The follow-up period for the first two clinical cases presented was for duration of 12 months, 18 months, 22 months, 24 months, and 22 months, respectively (Figures 8 [Fig fig9] [Fig fig10] [Fig fig11] [Fig fig12]–13 and 19 [Fig fig20] [Fig fig21] [Fig fig22]xref ref-type="fig" rid="fig23"/>–24). The patients were asymptomatic with clinical and radiological success when evaluated up to the respective follow-up intervals. The third clinical case was evaluated at the 2nd day postoperatively, 1st week, 3-month, 6-month, and 9-month intervals (Figures 29 [Fig fig30]–31). At the end of one year period, full coverage restorations, namely, porcelain fused metal crowns, were cemented as final definitive restorations. Crowns were placed after 12 months to ensure adequate favourable prognosis of the performed pulpotomy therapies.

**Figure 37:**
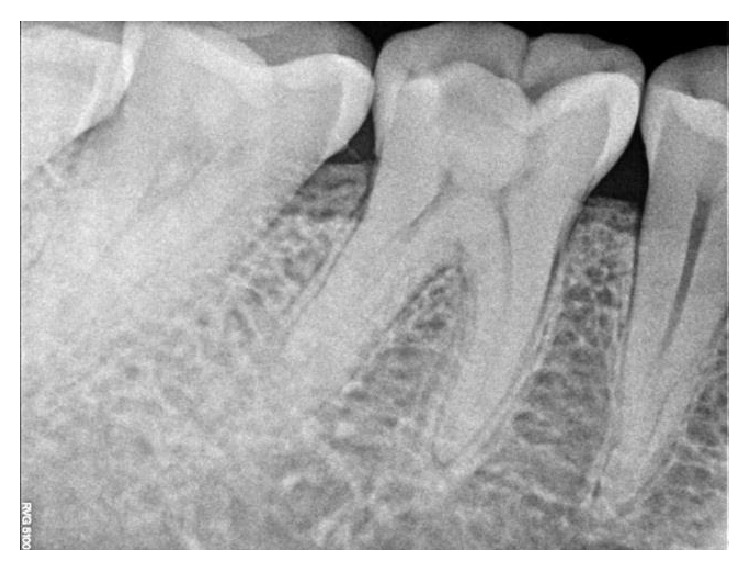
Preoperative RVG 36.

**Figure 38 fig38:**
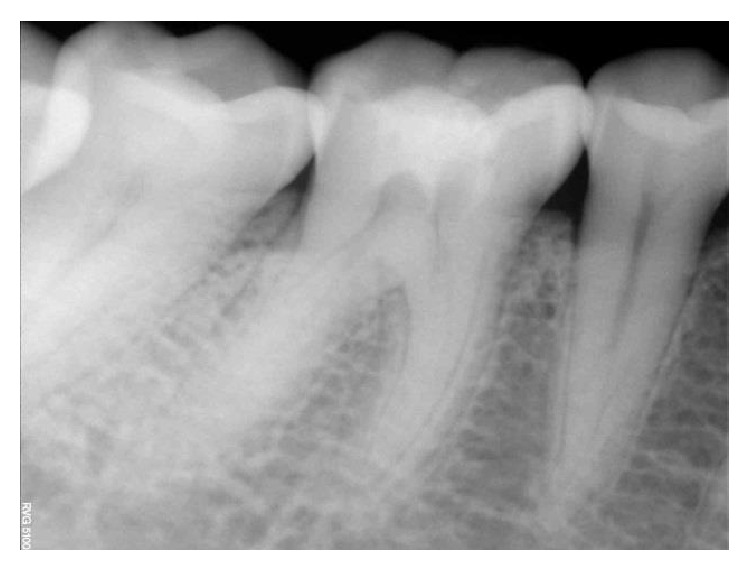
Immediate postoperative RVG 36.

**Figure 39 fig39:**
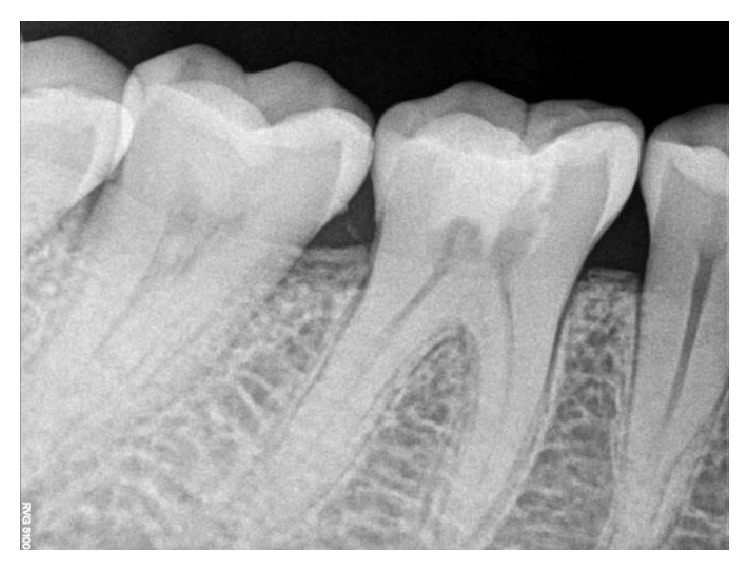
3-month followup RVG 36.

**Figure 40 fig40:**
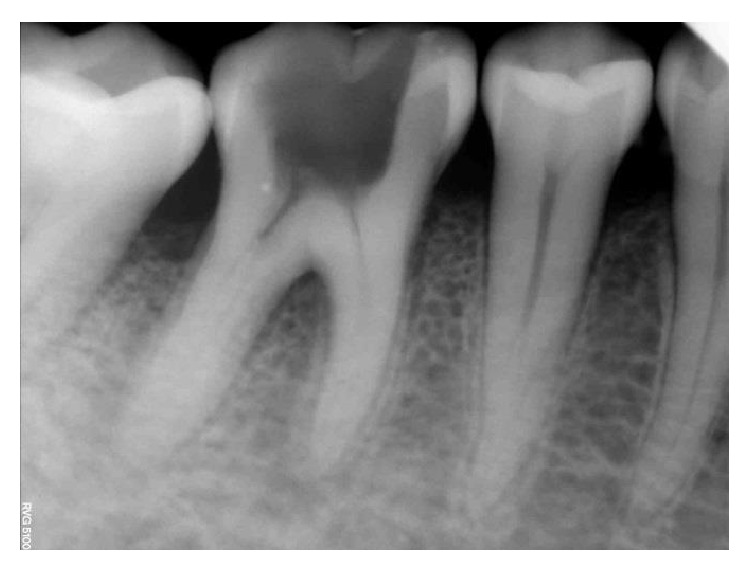
Radiograph showing the fracture of the cusp 36.

**Figure 41:**
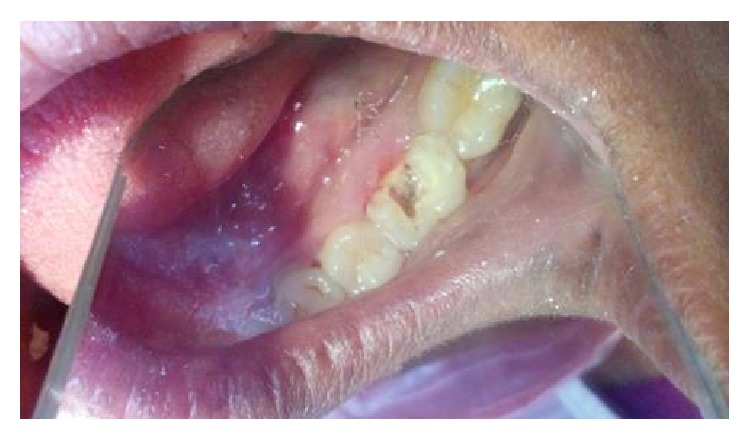
Photograph showing the fracture of the cusp 36 at the end of the 4-month interval.

However, in one of the four treated cases, we noticed a lingual crown fracture up to the level of the gingival third which breached the coronal seal, 3 months after the initiation of the treatment procedure (Figures 37 [Fig fig38] [Fig fig39] [Fig fig40]–41).   The patient gave a history of biting on a hard object during her meals using the tooth in question. She presented to our dental office only after one week after tooth crown fracture. During the restorative and periodontal evaluation of the involved tooth, we found that the coronal seal was lost and the initial planned pulpotomy procedure was discontinued as we had to perform the definitive treatment of root canal therapy.

The clinical follow-up evaluation of the remaining cases was met with a positive outcome. In addition, the digital radiographic examination (RVG) of the cases revealed an intact PDL space and a normal trabecular pattern of the bone. All the patients included in the clinical case series are still under rigorous systematic followup.

## 4. Discussion

The field of Endodontics has undergone numerous advances and new inventions in materials and techniques used for root canal treatment. With the introduction of nickel titanium rotary instruments, apex locators, operating microscopes, newer root end filling materials and devices, the quality of the endodontic treatment has drastically improved. Recent advances have broadened the scope of applications to provide the finest possible treatment and allow more teeth to be salvaged. However, root canal procedures in certain clinical situations still pose a challenge to the clinician, due to the myriad complexity of the root canal system and the complexities associated with the treatment procedures.

In the majority of the conditions, the primary cause leading to pulpal and periapical diseases is bacterial infection and the most common route of entry is through the carious process. The most reliable way of relieving the acute pain of a patient with irreversible pulpitis is by performing emergency treatments like pulpotomy or pulpectomy [10, 11].

When there are no restrictions on the time and the cost factor, root canal therapy can be an ideal choice of treatment in various clinical situations with a success rate of ±95% as evidenced in numerous literature studies [11]. However apart from being challenging in certain scenarios, it is relatively more time-consuming and expensive and often the outcome of the treatment provided by the general dentist is poor. Also patients from the lower economic strata opt for extraction of the involved tooth rather than the root canal therapy due to the cost factor associated with it. Therefore, alternative procedures such as pulpotomies could serve as viable, less invasive, potential treatment options and could help prevent unnecessary dental extractions or dental neglect in such situations [12].

Vital pulpotomy is basically considered as an emergency treatment procedure for the temporary relief of symptoms to reduce the swelling if present and to finally maintain the integrity of the tooth and arch in symptomatic irreversible pulpitis. The success rates of these procedures performed on primary teeth and on immature permanent teeth with open apices have been well documented. A clinical study was conducted by Kabaktchieva and Gateva on 4–8-year-old children in 33 primary molars, affected by the carious process, which revealed a 100% success rate of MTA pulpotomy after 6 months [13]. Another clinical investigation of similar nature was conducted by Nyerere et al. in 2006 in 180 patients over 15 years of age, with a chief complaint of pain associated with acute pulpitis. Pulpotomy was performed in molars or premolars of the selected patients using zinc oxide eugenol as the material of choice. Periodic evaluation at one, two, and six weeks demonstrated a success rate of 100% for premolars and 97.1% for molars in alleviating acute pain [14].

The procedure of pulpotomies in adult teeth with mature apices has been investigated to a much lesser extent and related controversies still exist in the literature. However, a systematic review conducted by Aguilar and Linsuwanont has demonstrated the success rate of vital pulp therapies in vital permanent teeth with closed apices, showing a relatively high success rate of 99.4% for partial pulpotomy and 99.3% for full pulpotomy [15]. Eghbal et al. have evaluated the histological success of pulpotomy in permanent molars of patients in the age ranging from 16 to 28 years and the histological observations revealed a complete dentinal bridge with radicular pulp remaining vital and free of inflammation in all the samples [3].

Various studies have reported the cytotoxicity of freshly mixed calcium silicate based synthetic materials because of their high initial pH. Hence in the present case series the radicular pulp tissue is covered with a biologically based material like PRF to avoid any detrimental effects on the pulp as a result of the synthetic cement materials [8].

Current research revolves around better scaffolds for use in regenerative endodontic treatment. Bezgin et al. aimed to clinically and radiographically evaluate the efficacy of platelet-rich plasma (PRP), 1st generation platelet concentrates, when used as a scaffold in regenerative endodontic treatment and compare it with that of a conventional blood clot (BC) scaffold. However, they concluded that the treatment outcomes did not differ significantly between both groups, though the PRP group performed better and showed faster healing [16].

PRF is an autologous source of the growth factors such as platelet derived growth factor (PDGF), transforming growth factor 1 (TGF *β*1) and insulin-like growth factor (IGF) [17]. It is a concentrate of platelets and cytokines widely employed to accelerate healing of the soft tissue and hard tissue lesions and is considered to be an ideal material to repair and regenerate the pulp-dentin complex [18].

Second generation blood matrices (PRF) which was used in the present case series is superior to platelet rich plasma (PRP) in various ways. Unlike PRP, the procedure for the preparation is simple and economical and the addition of bovine thrombin anticoagulants or the biomechanical handling of the blood is not required. By allowing slow polymerization, it helps in efficient migration, attachment, proliferation, and differentiation of the cells. It also provides support to the immune system and promotes hemostasis [19–21].

Various biomaterials have been introduced with the aim of safeguarding the vitality of the pulp. The prognosis of the treatment depends on the biocompatibility and the ability of the material to provide a good biological seal. However, one has to bear in mind that the ability of the pulp to respond to the injury also plays a significant role [20, 21].

Calcium hydroxide introduced by Herman has been the most commonly used biomaterial for pulpotomy. There are certain limitations associated with calcium hydroxide, such as its high initial pH which leads to the liquefaction necrosis of the superficial pulp tissue, its inability to bond to the dentin, and its noted dissolution over a period of time. Also it possesses poor mechanical properties and hence cannot prevent microleakage on the long run. In addition, the histological analysis of the hard tissue barrier formed using calcium hydroxide has a porous structure with tunnel defects which serve as portals of entry for microorganisms. This may eventually lead to secondary inflammation of the pulp and failure of the treatment [22].

MTA is considered to be a reliable alternative to calcium hydroxide in vital pulp therapy procedures due to its biocompatibility and improved sealing ability. It possesses good physical properties and provides an excellent marginal adaptation. It maintains a high pH for longer periods of time and stimulates reparative dentin formation at a faster rate than calcium hydroxide cement. Aeinehchi et al., in their clinical trial of eleven pairs of maxillary third molars in subjects between 20 and 25 years, demonstrated a dentinal bridge of 0.43 mm thickness with MTA versus that of 0.15 mm thickness with Ca(OH)_2_ when histological evaluation was done at 6 months of time [23]. It provokes less pulpal inflammation and the reparative dentin formed is thicker with fewer tunnel defects compared to calcium hydroxide. However, the drawback of MTA is its long setting time.

Based on the outstanding properties of MTA, another new bioactive calcium silicate based cement of similar composition with modified properties to improve the handling ability and to reduce the setting time was introduced as Biodentine (Septodont, Saint-Maur-des-Fossés, France). This material is advocated in clinical use as a biomaterial for procedures like pulp capping, pulpotomies, and so forth. Biodentine has also shown promise as a cervical lining restoration and may be utilized for the successful management of perforations and internal and external resorptive defects and apexification and retrograde filling [24]. It also shows improved mechanical properties and reduced setting time of 12 min. The advantage of using Biodentine in the present case series is that it is biocompatible and insoluble, has good mechanical properties, and provides a tight biological seal against the ingress of bacteria [25].

Calcium hydroxide or other calcium silicate based materials mainly increase the release of TGF-Beta1 from the dentin or from the pulp tissue. The results obtained in an in vitro study in which the dental pulp cells treated with TGF-Beta1 differentiated into odontoblast-like cells expressing dentin proteins such as dentin sialophosphoprotein and dentin matrix protein-1 [26, 27]. TGF-Beta1 promotes progenitor cell migration and promotes odontoblast differentiation, mineralization, and regeneration of the pulp. Odontoblast differentiation and initiation of mineralization were enhanced with calcium silicate based materials when compared with calcium hydroxide because of the presence of both calcium and silicon ions [26].

Zanini et al. also evaluated the biological effect of Biodentine on murine pulp cells by studying the expression of several biomolecular markers after culturing OD-21 cells with or without Biodentine. Their results, being consistent with other studies, were in favor of Biodentine, which was found to be bioactive due to its ability to increase OD-21 cell proliferation and biomineralization [28].

MTA may induce pulp healing with dentin bridge formation and prevent necrosis at long-term periods in most of the pulpotomy cases. However, discoloration following MTA pulpotomy is a significant clinical complication [29, 30]. One study evaluated the pulpal and periapical responses of dogs' teeth after pulpotomy and pulp capping with a new tricalcium silicate based cement (Biodentine) when compared with mineral trioxide aggregate (MTA) by radiographic, histopathologic, and histomicrobiological analyses. The authors observed that Biodentine presented tissue compatibility and allowed for mineralized tissue bridge formation after pulpotomy with similar morphology and integrity to those formed with use of MTA [31].

Villat et al. performed partial pulpotomy using Biodentine in an immature second right mandibular premolar and demonstrated a fast tissue response both at the pulpal and root dentin levels. They suggested that the use of tricalcium silicate cement should be considered as a conservative intervention in the treatment of symptomatic immature teeth [32, 33].

In the present case series, Biodentine was filled using a single stage approach where the Biodentine was placed in contact with the pulp tissue and it was allowed to set for 12–15 mins and followed by the permanent restoration on the top of it. Two-stage approaches can also be followed where the entire cavity is filled with Biodentine and is then reduced to a base or substrate level for 1 week to 6 months later for the permanent restoration. But it is associated with certain drawbacks; namely, it requires the compliance of the patient, unavoidable cavity preparation at second appointment which may expose the pulp tissue, excessive pressure during trimming, and polishing which may disrupt the crystalline structure and lead to the loss of the marginal strength of the material.

The PRF obtained from the patient is squeezed to produce a membrane and placed over the radicular pulp. Biodentine helps to stabilize and protect the membrane from the compaction forces of the restorative procedures amongst the other advantages. Taking this into consideration, a layer of Biodentine was placed over the PRF membrane.

The idea behind the pulpotomy therapy was to provide a good biological seal, because if further entry of bacteria is prevented into the exposed pulp tissue, it has an ability to heal with the formation of new dentinal bridge and the periapical tissues undergo regeneration. The success of the pulpotomy procedure depends on the right choice of the biomaterial in terms of its biocompatibility, sealing ability, ability to stimulate reparative dentin formation, and regenerative potential of the pulp.

During the treatment procedure, the pulp should be free from bacteria and its toxins and preventing the invasion of bacteria into the pulp is the vital factor for favourable prognosis of this procedure. It can be achieved using rubber dam isolation which prevents the invasion of bacteria from the oral cavity and saliva. Provision of the double seal also prevents the bacterial leakage.

Biodentine can be considered as a restorative material of choice because of high mechanical properties and good sealing ability. Hence, in the present case series, double seal provided by Biodentine and composite restoration ensures a tight coronal seal [34].

The bleeding induced during the removal of the pulp tissue should be rapidly controlled. Proper hemostasis is indispensable because the blood clots that remain at the pulp material interface leads to treatment failure. The ideal solution for hemostasis is 0.5% of sodium hypochloride because it helps in rapidly controlling the bleeding and disinfecting the cavity.

The age of the patient is important criteria for the selection of the patients with vital pulp therapies because older pulps are fibrous and less cellular with limited blood supply affecting the treatment outcome [2]. Taking into consideration the direct impact of age on the success rate of such procedures, pulpotomy was planned for all the 4 selected patients with age ranges between 17 and 22 years to achieve a predictable outcome.

## 5. Conclusion

In the present case series, the advantages of the growth factor releasing potential of PRF and sealing ability of Biodentine are utilized as a double edged sword to accelerate healing of the irreversible inflamed pulp tissue as an alternative to extirpation of the same.

The probable reasons attributed to the high success rates of the cases presented (four out of the five documented clinical cases) could be in accordance with the findings of the various scientific studies which states that the bacteria gain access to the pulp lumen only after a considerable part of the pulp has been involved.

Apart from the chosen regenerative materials of choice, the age, general health, diagnostic criterion, oral hygiene practices, economics, patient motivation, and compliance were other important factors which were focused on during the case selection while opting for pulpotomy modality of treatment over conventional Endodontics.

Other contributing factors towards the success of the treatment performed include strict aseptic protocols, rapid coverage of the exposed pulp stumps, appropriate regenerative scaffold, and a bacterial tight coronal double seal.

Clinician's interest, skill, intuition, and knowledge play a crucial role in the art of decision making to provide conservative, viable, and safe treatment alternatives such as pulpotomies over pulpectomies in irreversibly inflamed adult permanent teeth with closed apices. Within the limits of our present clinical study and based on the positive outcomes achieved in the systematic follow up case series, we can conclude that clinicians can safely rely upon advanced non-invasive, regenerative approaches to improve the standard of care delivered to the patients. However further studies and clinical trials on the effectiveness of such procedures are still required to consider it as a main stay of treatment.

## Figures and Tables

**Figure 14:**
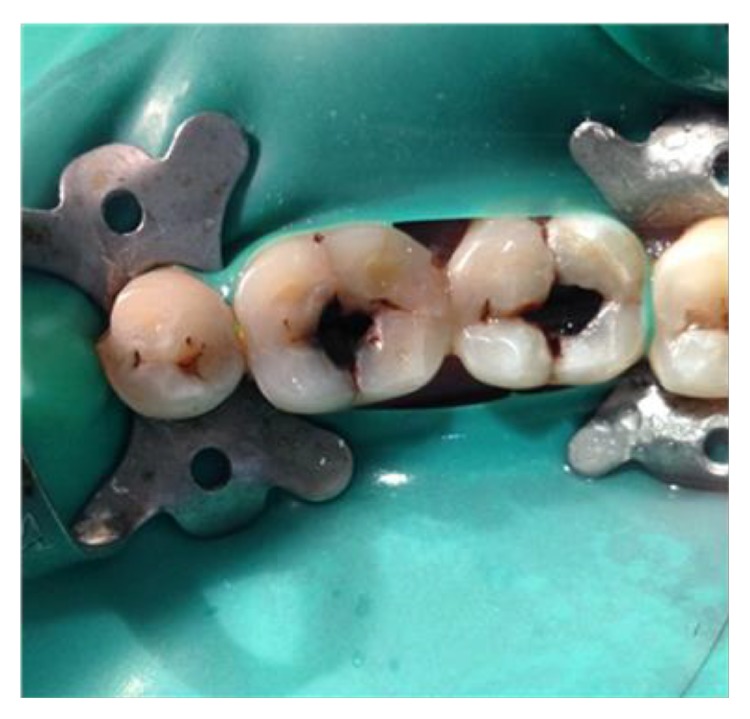
Preoperative photograph: 36, 37.

**Figure 15 fig15:**
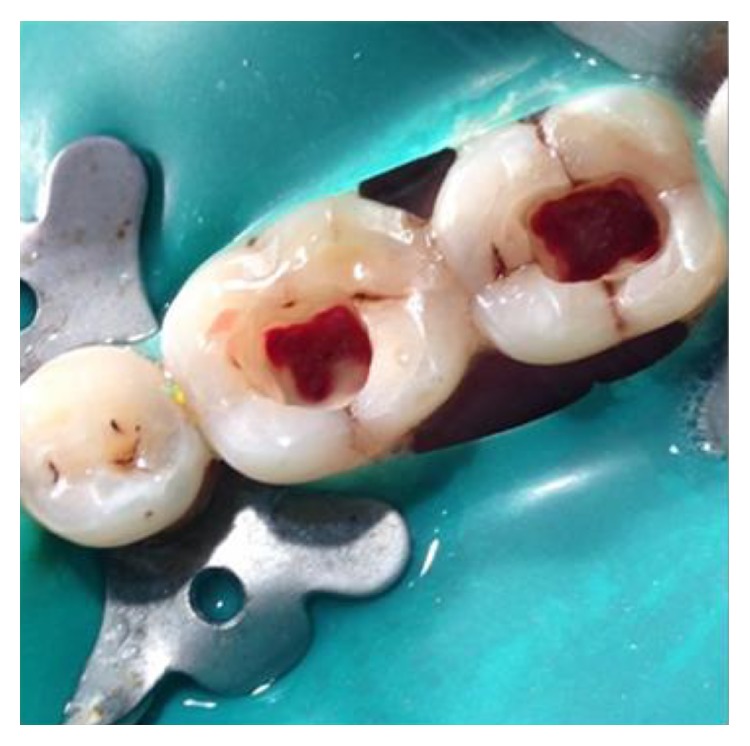
Access opening for pulpotomy procedure: 36, 37.

**Figure 16 fig16:**
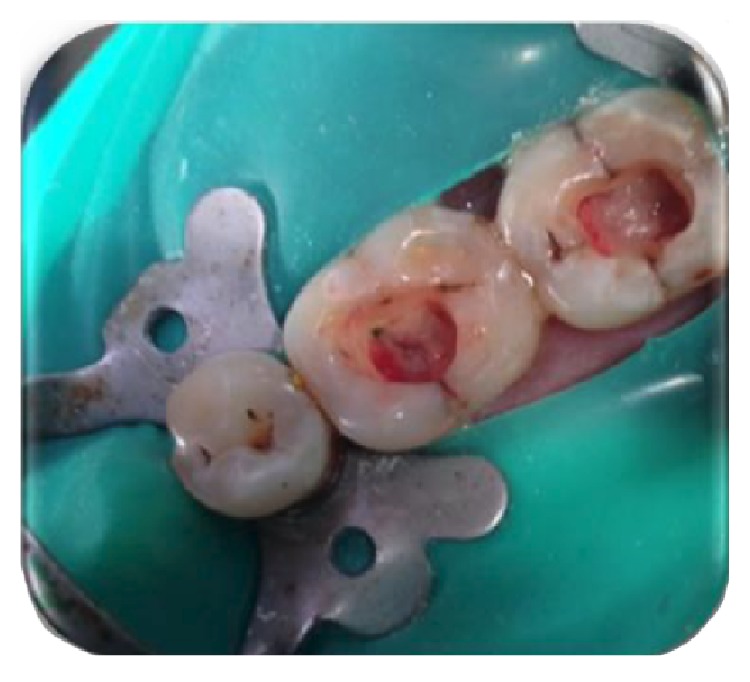
Placement of PRF membrane over the radicular pulp: 36, 37.

**Figure 17 fig17:**
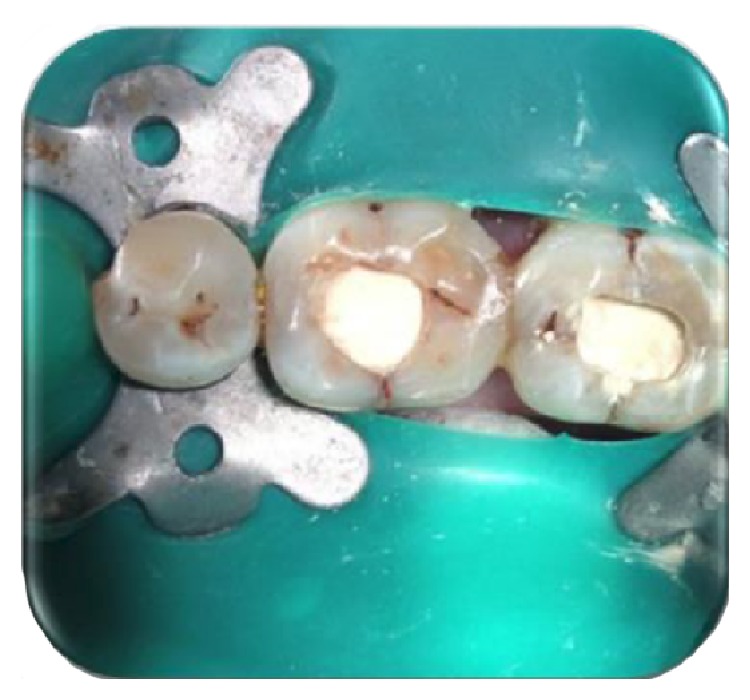
Placement of Biodentine over PRF: 36, 37.

**Figure 18:**
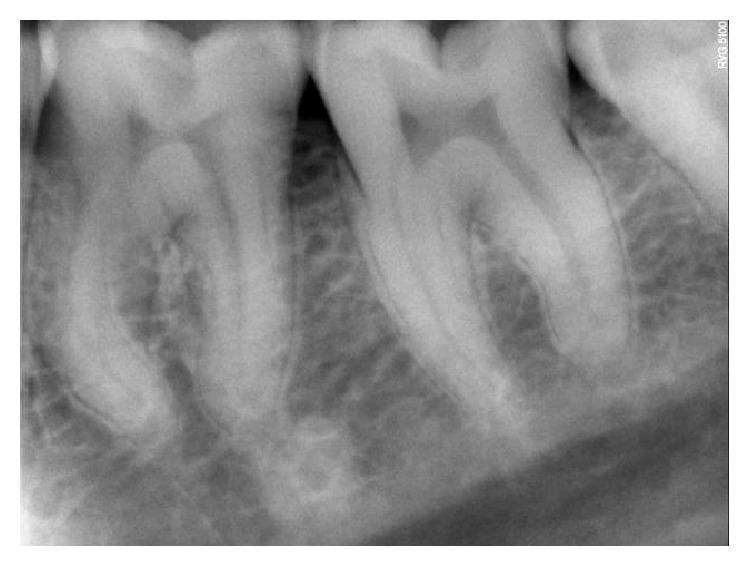
Preoperative RVG: 36, 37.

**Figure 24:**
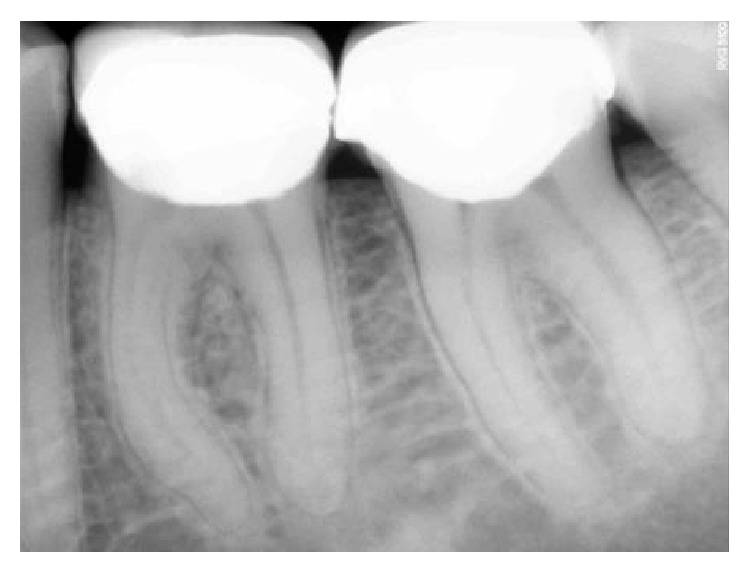
22-month followup RVG: 36, 37.

**Figure 25:**
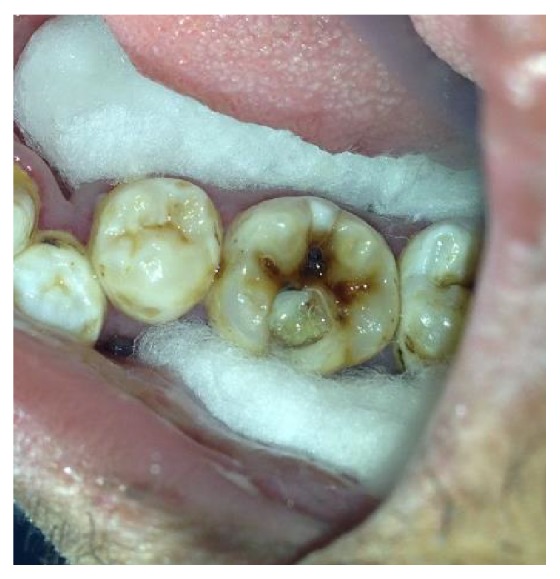
Preoperative photograph 36.

**Figure 26 fig26:**
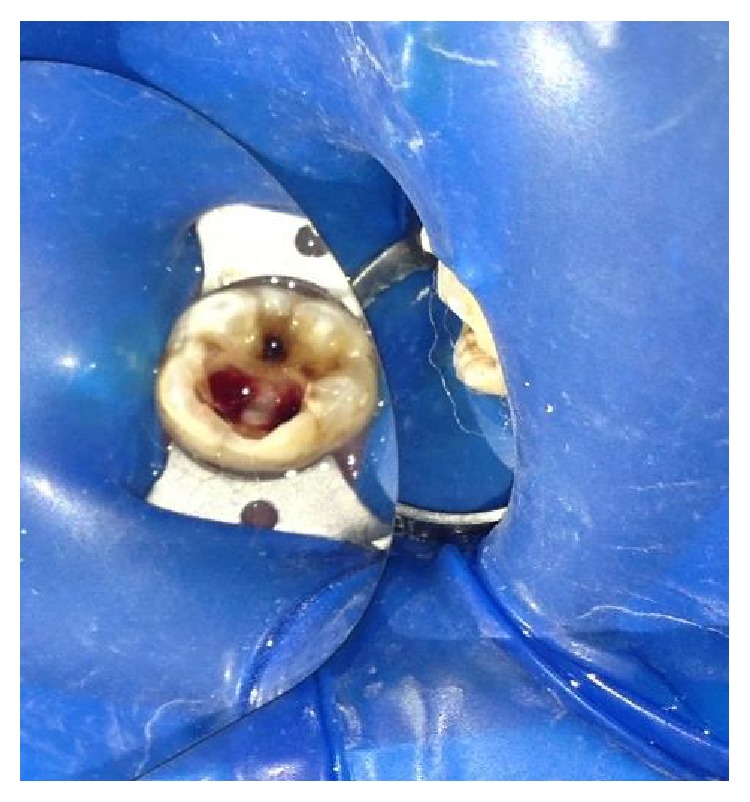
Access opening for pulpotomy procedure 36.

**Figure 27 fig27:**
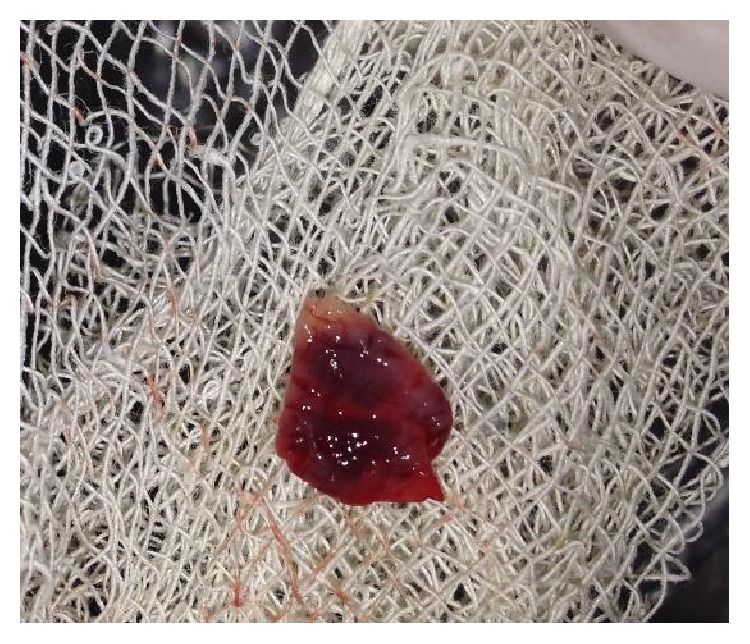
PRF membrane 36.

**Figure 28:**
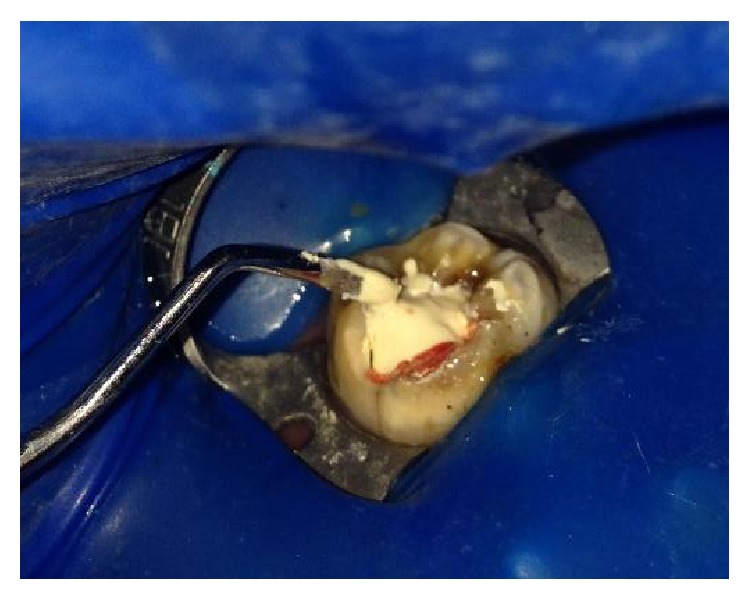
Placement of PRF and Biodentine over the radicular pulp 36.

**Figure 32:**
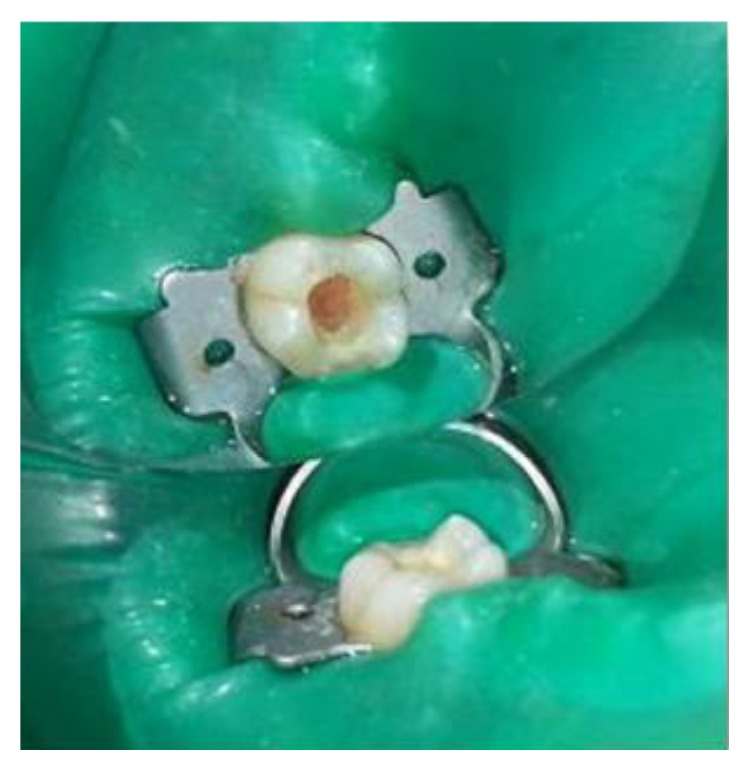
Preoperative photograph 36.

**Figure 33 fig33:**
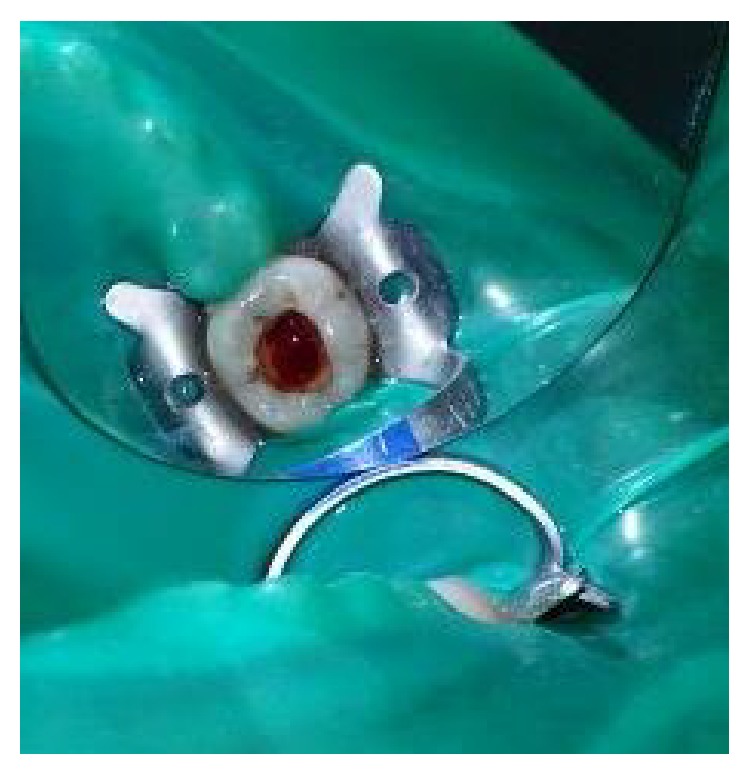
Access opening for pulpotomy procedure 36.

**Figure 34 fig34:**
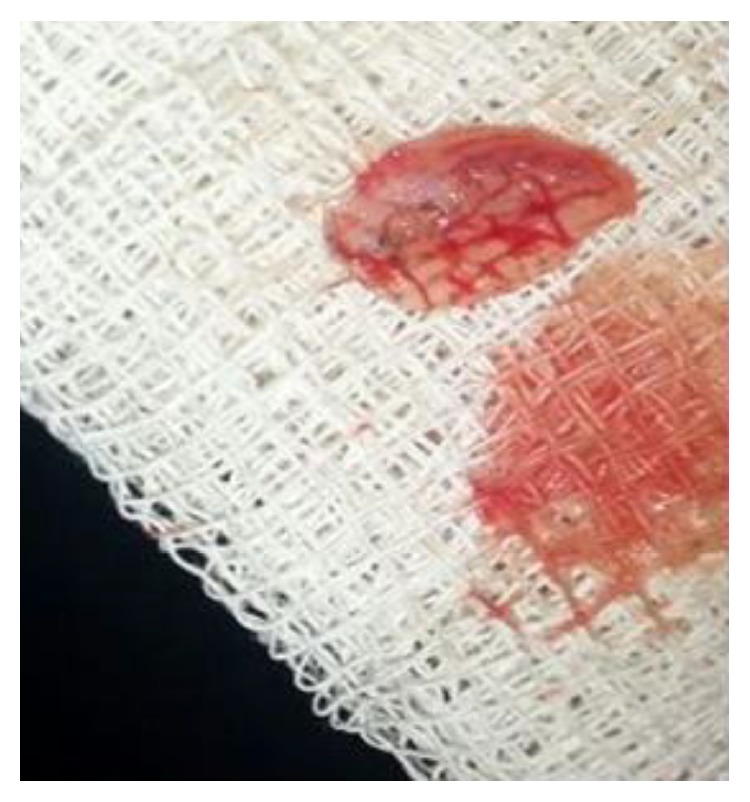
Platelet rich fibrin membrane.

**Figure 35 fig35:**
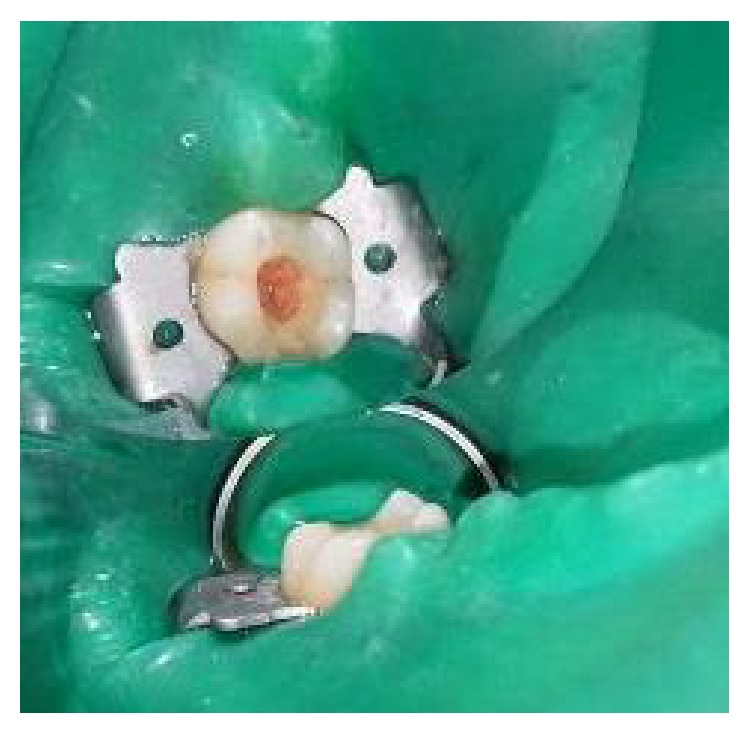
Placement of PRF membrane over the radicular pulp 36.

**Figure 36:**
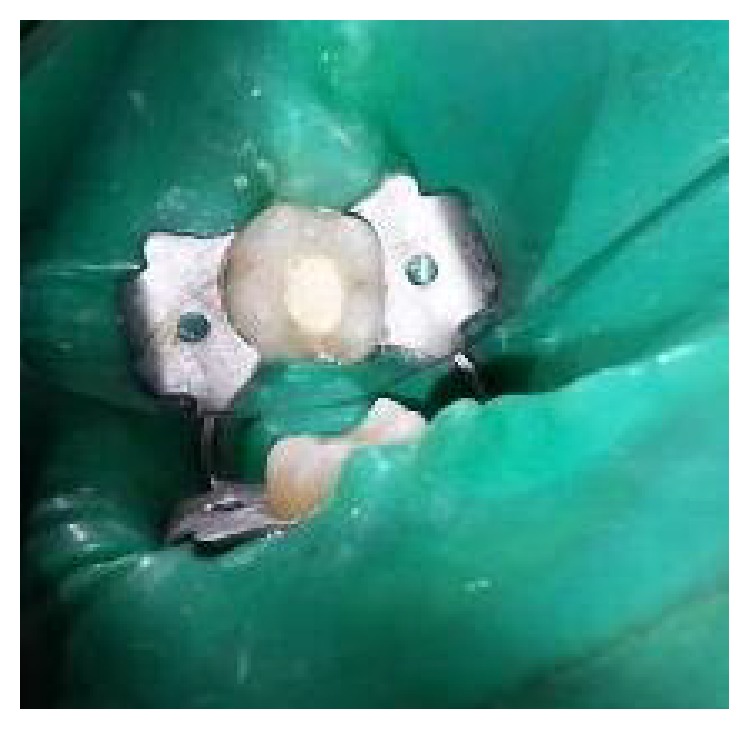
Placement of Biodentine over the PRF membrane 36.
